# Advances in Statistical Medicine

**DOI:** 10.1155/2014/316153

**Published:** 2014-11-09

**Authors:** Sujay Datta, Xiao-Qin Xia, Samsiddhi Bhattacharjee, Zhenyu Jia

**Affiliations:** ^1^Department of Statistics, University of Akron, 302 Buchtel Commons, Akron, OH 44325, USA; ^2^Institute of Hydrobiology, Chinese Academy of Sciences, No. 7 Donghu South Road, Wuhan, Hubei 430072, China; ^3^National Institute of Biomedical Genomics, Netaji Subhas Sanatorium, 2nd Floor, P.O. Box N.S.S., Kalyani, West Bengal 741251, India; ^4^Department of Family and Community Medicine, Northeast Ohio Medical University, 4209 Ohio 44, Rootstown, OH 44272, USA

We are not even halfway through the second decade of the 21st century and there is already ample evidence that it is going to be the century of biotechnology, leading to unprecedented breakthroughs in the medical sciences and revolutionizing everything from drug discovery to healthcare delivery. The rapid advancement in high-performance computing that took place in the last quarter of the last century has been a key driving force in this revolution, enabling us to generate, store, query, and transfer huge amounts of medical data. This is where statisticians come into the picture, lending their expertise in extracting information from data and converting that information to medical knowledge.

The crucial role that statisticians have been playing in this information revolution has created new challenges and posed difficult problems for their own discipline. Dealing with them has often necessitated new statistical techniques, new approaches to inference, or even new modes of thinking. These, in turn, have been the motivating force behind an astonishing flurry of biostatistical research activities in the recent years. In the ten carefully chosen and peer-reviewed articles of this special issue, we hope to provide a nuanced perspective on some of the areas in the biomedical sciences that have directly benefited from that research. This thriving partnership between experts in the quantitative world and those in the medical world has been highly interdisciplinary in nature. This special issue aims to introduce researchers, practitioners, and students on both sides of the fence to some of the statistical modeling and inference approaches that have collectively had such a huge impact on the field of medicine. And there is a clear need for it.

Due to the injection of a steady flow of new technologies, the medical field has progressed rapidly and has produced data at a phenomenal rate. It is important for those in the medical world to understand that the types of data collected and the manner in which they are collected are crucial to the validity and reliability of the subsequent statistical analysis. Some basic familiarity with statistical methodologies will make them aware of the potential pitfalls of some designs of experiments in certain contexts and enable them to choose better ones. Also, they need to realize that statistical analysis is not a mechanical process like solving a set of mathematical equations. Specifying a statistical model that is appropriate for a given situation and drawing conclusions about the model parameters are fraught with many challenges. This realization will give them a better appreciation of the role that a statistician plays in medical research. On the other hand, statisticians will be motivated to develop methodologies capable of handling systems that change constantly with time and in response to therapeutic, physiological, and environmental stimuli. They will see the need for dealing with mathematical models that are much more complex and challenging than those routinely encountered in the rest of statistics.

The articles in this special issue were chosen with this in mind. J. V. Pottala et al. use a latent variable approach and structural equation modeling for analyzing erythrocyte fatty acids in the context of the Framingham study. B. Miller et al. use chi-squared automatic interaction detection decision trees and waist circumference as a surrogate measure to detect metabolic syndrome in young adults. M. Banerjee et al. use logic regression in an innovative way in the context of kidney cancer treatment delivery to uncover the complex interplay among patient, provider, and practice environment variables based on linked data from the National Cancer Institute's Surveillance, Epidemiology and End Results Program and Medicare. Z. Huang and Y. Chen propose and implement a two-stage model based on synergetic neural networks for exon recognition, a fundamentally important task in bioinformatics. J. A. Koziol and Z. Jia generalize the quadratic version of the log-rank test, introduced originally by Lin and Wang, to incorporate weights that increase statistical power in some situations. H. Li et al. construct an association network between micro-RNA and cancer based on more than a thousand miRNA-cancer associations detected from millions of abstracts using a text-mining method. C. Taslim and S. Lin propose a mixture modeling framework that is flexible enough to automatically adapt to most high-throughput data-types that are encountered in modern genomics, thereby overcoming the difficulty that statistical methods specifically designed for one data-type may not be optimal for or applicable to another data-type. P. T. Edlefsen shows through examples that the heterogeneous effects of leaky vaccines (that protect subjects with fewer exposures to a pathogen at a higher effective rate than subjects with more exposures) violate the proportional hazards assumption, leading to incomparability of infected cases across treatment groups and to nonindependence of the distributions of the competing failure processes in a competing risks setting. E. Cheng and Z. M. Ozsoyoglu propose a framework for deriving path-counting formulas for all generalized kinship coefficients for which there are recursive formulas and which are sufficient for computing condensed identity coefficients, an important computation on Pedigree data that provides a complete description of the degree of relatedness between two individuals. Finally, R. L. Einsporn and Z. Jia shed the light on some problems with interaction and varying block sizes in a comparison of endotracheal tubes through a randomized clinical experiment based on a block design.

Collectively, the editors express their sincerest gratitude to their respective institutions for the time and resources that they were provided. And last but not least, the editors gratefully acknowledge the support and encouragement they received from their respective families during this endeavor.

*Sujay Datta* *Sujay Datta* *Xiao-Qin Xia* *Xiao-Qin Xia* *Samsiddhi Bhattacharjee* *Samsiddhi Bhattacharjee* *Zhenyu Jia* *Zhenyu Jia*

